# Extensions of BLUP Models for Genomic Prediction in Heterogeneous Populations: Application in a Diverse Switchgrass Sample

**DOI:** 10.1534/g3.118.200969

**Published:** 2019-01-16

**Authors:** Guillaume P. Ramstein, Michael D. Casler

**Affiliations:** * Department of Agronomy, University of Wisconsin-Madison, Madison, WI 53706; † Agricultural Research Service, United States Department of Agriculture, Madison, WI 53706

**Keywords:** Genomic Prediction, population heterogeneity, marker-by-population interaction, kernel functions, *Panicum virgatum*, GenPred, Shared Data Resources

## Abstract

Genomic prediction is a useful tool to accelerate genetic gain in selection using DNA marker information. However, this technology typically relies on standard prediction procedures, such as genomic BLUP, that are not designed to accommodate population heterogeneity resulting from differences in marker effects across populations. In this study, we assayed different prediction procedures to capture marker-by-population interactions in genomic prediction models. Prediction procedures included genomic BLUP and two kernel-based extensions of genomic BLUP which explicitly accounted for population heterogeneity. To model population heterogeneity, dissemblance between populations was either depicted by a unique coefficient (as previously reported), or a more flexible function of genetic distance between populations (proposed herein). Models under investigation were applied in a diverse switchgrass sample under two validation schemes: whole-sample calibration, where all individuals except selection candidates are included in the calibration set, and cross-population calibration, where the target population is entirely excluded from the calibration set. First, we showed that using fixed effects, from principal components or putative population groups, appeared detrimental to prediction accuracy, especially in cross-population calibration. Then we showed that modeling population heterogeneity by our proposed procedure resulted in highly significant improvements in model fit. In such cases, gains in accuracy were often positive. These results suggest that population heterogeneity may be parsimoniously captured by kernel methods. However, in cases where improvement in model fit by our proposed procedure is null-to-moderate, ignoring heterogeneity should probably be preferred due to the robustness and simplicity of the standard genomic BLUP model.

Genomic prediction has proved a useful tool to predict genetic merit in plant and animal breeding (Hayes *et al.* 2009a, Lorenz *et al.* 2011). This technology consists of learning relationships between DNA markers and phenotypes, which arise from the non-random association (linkage disequilibrium; LD) between DNA markers and causal genetic variants having direct effects on the trait studied (Meuwissen *et al.* 2001). Standard genomic prediction models, including genomic BLUP (GBLUP; VanRaden 2008, Hayes *et al.* 2009b) or Bayesian linear regression (BLR) models (Meuwissen *et al.* 2001, Gianola *et al.* 2009), assume that the effects of causal variants are linear and purely additive, so estimated effects do not capture any dependence on context, arising for example from interactions of causal variants with environmental or genetic backgrounds. Initially, genomic prediction models have been proposed for applications in populations that are relatively homogeneous with respect to LD patterns and interactions involving causal variants (Meuwissen *et al.* 2001). In such situations, increasing the size of the calibration set (CS) – the set of individuals used to estimate the model’s parameters – would typically benefit accuracy of the models (Lorenzana & Bernardo 2009, VanRaden *et al.* 2009). However, in practice, increasing the CS size may often involve calibrating prediction models on individuals with inconsistent LD patterns and/or backgrounds, which may result in reduced accuracy (Wientjes *et al.* 2016). This issue will arise in the typical situation where an initially homogeneous CS is augmented with individuals from extraneous populations, that is, multi-population – or (in the animal literature) multi-breed – calibration (Lund *et al.* 2014). Recently, studies in both plant and animal breeding have assessed the usefulness of combining populations from different genetic backgrounds in genomic prediction.

Under standard genomic prediction models (where population heterogeneity is ignored), the simulation study of de Roos *et al.* (2009) suggested that adding an extraneous population to a CS may benefit prediction accuracy if the added population is not too dissimilar (in terms of divergence time) from the initial CS. These authors also suggested that high enough marker density could prevent prediction accuracy from decreasing, even in cases of strong divergence between populations. Consistently, most empirical studies of multi-population calibration with high marker density, based on standard GBLUP and/or BLR, have reported little or no gain in accuracy under strong population structure (Lehermeier *et al.* 2015, Jarquin *et al.* 2016, Hayes *et al.* 2009c, Erbe *et al.* 2012). In contrast, only a few studies have reported substantial increases in accuracy from multi-population calibration in similar conditions (Technow *et al.* 2013, Daetwyler *et al.* 2012).

In multi-population prediction models (where marker-by-population interactions are explicitly accounted for), studies have proposed to fit, to the whole set of available individuals, models that were capable of accommodating population heterogeneity explicitly. This type of models includes multi-trait GBLUP models, with “traits” corresponding to population backgrounds (Karoui *et al.* 2012, Carillier *et al.* 2014, Lehermeier *et al.* 2015), and random regression models based on markers interacting with discrete population cluster coefficients (de Los Campos *et al.* 2015, with an extension of a standard BLR model). To our knowledge, the implementation of these methods has not been adapted to contexts of admixture, where population structure variables are continuous. Furthermore, when calibration involves many populations, the increase in model complexity of these methods will make them computationally intractable and statistically inefficient. Parsimonious multi-population models, based on only a few parameters to capture population heterogeneity, have also been proposed (Zhou *et al.* 2014, Heslot and Jannink 2015). In the presence of many populations, such models are more practical and potentially more useful than multi-trait and random interaction models. Also, since they generally assume some underlying basis for population heterogeneity (*e.g.*, inconsistency in LD patterns), they may generate insight about the causes of marker-by-population interactions.

In this study, we first considered a standard GBLUP model and evaluated different types of fixed effects to reflect population structure, then we investigated the usefulness of standard GBLUP and two kernel-based extensions for coping with population heterogeneity. These procedures were compared to a standard GBLUP model under two validation schemes: whole-sample calibration, where all individuals except selection candidates are included in the calibration set, and cross-population calibration, where the target population is excluded from the calibration set. The two multi-population GBLUP extensions are one previously-reported model, derived from Heslot and Jannink (2015), and one proposed model, based on a flexible kernel function of population principal components. We applied the procedures to the analysis of three traits (plant height, heading date, and standability) in switchgrass (*Panicum virgatum* L.), an herbaceous biomass crop showing good promise for bioenergy production (Sanderson *et al.* 1996, Langholtz *et al.* 2016). This species is characterized by an extensive diversity which makes it particularly suitable for studying population heterogeneity (Casler 2012). The sample under study comprised seven population clusters from two diverse panels, assayed in the Midwestern region of the United States, which represent differentiation by ecotype (upland or lowland), geographical origin (latitudinal and longitudinal gradients) and ploidy level (tetraploid or octoploid). This sample exemplified the heterogeneity of data available for practical applications of genomic prediction, which pose both opportunities (by increased sample sizes) and challenges (by inconsistencies in marker effects across populations) for breeding based on DNA markers.

In this study, we did not fit BLR models (usually based on Markov chain Monte Carlo optimizations), since we focused on deterministic methods for model fit and considered only models based on computationally efficient best linear unbiased predictors (BLUP). Further research would be needed to develop kernel-based extensions of this type of models.

## Material and methods

### Panels and populations

In this study, two multi-population panels were assayed and considered together in one sample. The first panel was the breeding panel (BP) described in Ramstein *et al.* (2016), comprising two tetraploid breeding populations of half-sib families: WS4U-C2, which consisted of 137 half-sib families derived from a diverse upland-ecotype pool of 162 plants (Casler *et al.* 2006), and Liberty-C2, which consisted of 110 half-sib families derived from the lowland-upland cultivar Liberty (Casler and Vogel 2014). The second panel was the association panel (AP) described in Lu *et al.* (2013) and Evans *et al.* (2015), comprising six putative populations of clonally propagated genotypes of different ecotypes (U: upland; L: lowland), ploidy levels (4X: tetraploid; 8X: octoploid) and geographical origins (S: South; W: West; N: North; E: East): U4X-N (135 plants), U8X-W (129 plants), U8X-E (97 plants), U8X-S (10 plants), L4X-NE (106 plants) and L4X-S (37 plants). These populations corresponded to 66 diverse accessions (Lu *et al.* 2013, Evans *et al.* 2015) with up to 10 individuals per accession.

In WS4U-C2, one individual was discarded so as to avoid assigning it to a population in AP, since it was too distantly related to the other individuals in BP (based on principal component analysis). In total, n=760 individuals were considered in this analysis. The main goal of this study was to assess different methods for accommodating genetic heterogeneity when predicting phenotypic means in a given target population. Four targets were chosen, with a defined focus on tetraploid populations with at least 100 relatively homogeneous individuals: WS4U-C2 and Liberty-C2 (from BP), and U4X-N and L4X-NE (from AP).

### Marker data

Exome capture sequencing of individuals (parents in BP and clonally propagated plants in AP) was performed using the Roche-Nimblegen protocol for preparation of SeqCap EZ Developer libraries using the Roche-Nimblegen probeset ‘120911_Switchrass_GLBRC_R_EZ_HX1’ as described previously (Evans *et al.* 2014, 2015). Reads from sequencing were aligned to the hardmasked *P. virgatum* v1.1 reference genome (http://phytozome.jgi.doe.gov/pz/portal.html#!info?alias=Org_Pvirgatum). Counts of reads corresponding to alternate and reference alleles for each individual were then determined as described previously (for BP, Ramstein *et al.* 2016; for AP, Evans *et al.* 2014, 2015) at 2,179,164 single nucleotide polymorphism (SNP) loci, which were identified as polymorphic in two diversity panels: the Northern Switchgrass Panel, corresponding to AP (Evans *et al.* 2014, 2015), and a southern switchgrass panel (E. C. Brummer, unpublished data). The numbers of alternate allele at the SNP loci were then called by using the expectation-maximization algorithm of Martin *et al.* (2010) fitted in each population (in BP) or accession (in AP) separately, under the assumption of disomic inheritance. Although this assumption is supported in switchgrass for tetraploid genotypes (Okada *et al.* 2010; Li *et al.* 2014), it does not hold for octoploid genotypes, which would presumably exhibit tetrasomic inheritance. However, we did not adapt the algorithm of Martin *et al.* (2010) to accommodate possible tetrasomic inheritance, as the sequencing depth of ∼24 was deemed insufficient for calling intermediate heterozygotes (simplex and triplex) with high enough accuracy (Evans *et al.* 2015). Indeed, a sequencing depth of at least 60-80 was recommended by Uitdewilligen *et al.* (2013) for accurately calling tetrasomic genotypes. Therefore, for all individuals, the resulting marker-data matrix consisted of expected allelic dosages (sums of alternate-allele counts weighted by their posterior probabilities, for every individual and SNP) between 0 and 2.

The SNP data were filtered based on the following criteria: (i) proportion of missing values strictly lower than 2%, a stringent threshold given prior filtering of SNPs on read depth ≥ 5 (Evans *et al.* 2014, 2015); (ii) minor allele frequency strictly greater than $\frac{1}{2n}$ and variance of allelic dosages strictly greater than $2\left(\frac{1}{2n}\right)\left(1-\frac{1}{2n}\right)$, with n=760 individuals; (iii) *p*-value for Hardy-Weinberg equilibrium strictly greater than 10^−4^ in each BP population; (iv) availability of genomic-location information (as per v1.1 of the reference genome of *P. virgatum*). Missing values at SNPs were imputed by their mode in the whole sample. The resulting n×m filtered and imputed marker-data matrix **X** consisted of allelic dosages at m= 717,814 SNP markers.

### Phenotypic data

Populations in BP were assayed each year between 2012 and 2014, in Arlington, WI (USA), in a randomized complete block design, with four replicates for WS4U-C2 and three replicates for Liberty-C2. Populations in AP were assayed each year between 2009 and 2011 in Ithaca, NY (USA), in a sets-in-reps design, with two replicates per individual and 10 sets within each replicate, with each set comprising at most one individual from each of the 66 accessions in AP (Lu *et al.* 2013, Evans *et al.* 2015). In each panel, three phenotypic traits were considered: plant height, heading date and standability. Plant height (PH) was measured in centimeters as the height from the ground to the top of the tallest panicle. Heading date (HD) was measured in growing degrees days as the cumulated sum of daily average temperatures (in degrees Celsius; °) above 10 °, from January 1^st^ to the day of heading, defined as the emergence of at least half of the panicles from the boot (Mitchell *et al.* 1997); daily average temperatures were estimated by the average of the minimum and maximum daily temperatures. Standability (St) was measured on a 0-10 scale to describe plants’ stature and stiffness, with 0 qualifying plants that are prostrate and 10 qualifying upright and rigid plants (Lipka *et al.* 2014).

Not all traits were measured every year in any given population: only HD was measured in all three years in AP populations and Liberty-C2. For all other cases, measurements were available for only a subset of years (Table 1).

**Table 1:** Description of populations and trait measurements

Pop.	Size	Loc.	Trait	Years	Mean	Range
**L4X-NE**	106	NY	PH	2009 2011	142.9	95.8 - 205.2
			HD	2009 2010 2011	547.1	422.9 - 810.4
			St	2010 2011	5.6	1.0 - 8.9
**L4X-S**	37	NY	PH	2009 2011	209.7	130.7 - 240.1
			HD	2009 2010 2011	841.3	711.2 - 1075.6
			St	2010 2011	7.1	5.0 - 9.7
**Liberty-C2**	110	WI	PH	2012 2013	185.6	133.9 - 239.9
			HD	2012 2013 2014	806.3	652.1 - 979.7
			St	2013	6.2	2.7 - 8.9
**U4X-N**	135	NY	PH	2009 2011	155.5	93.7 - 207.7
			HD	2009 2010 2011	534.3	345.4 - 904.0
			St	2010 2011	5.4	1.6 - 8.2
**WS4U-C2**	136	WI	PH	2012 2013	163.8	127.9 - 204.6
			HD	2013 2014	527.6	405.7 - 692.4
			St	2013	5.7	2.0 - 8.2
**U8X-E**	97	NY	PH	2009 2011	168.2	101.0 - 225.2
			HD	2009 2010 2011	530.4	408.5 - 734.7
			St	2010 2011	5.6	1.7 - 8.0
**U8X-W**	129	NY	PH	2009 2011	165.2	124.7 - 224.7
			HD	2009 2010 2011	608.0	429.2 - 823.1
			St	2010 2011	3.5	0.7 - 7.2
**U8X-S**	10	NY	PH	2009 2011	175.4	138.7 - 190.8
			HD	2009 2010 2011	716.6	569.9 - 859.1
			St	2010 2011	5.8	4.0 - 7.5

Population (Pop.): WS4U-C2 is a collection of upland ecotypes; Liberty-C2 is a cross between upland and lowland ecotypes; other populations are designated by ecotype (U: upland; L: lowland), ploidy level (4X: tetraploid; 8X: octoploid) and geographical origin (S: South; W: West; N: North; E: East). Location (Loc.): location of phenotypic trials, Arlington (WI, USA) or Ithaca (NY, USA). Trait: plant height (PH), heading date (HD) or standability (St). Mean and range refer to the means *y_i_*’s as described in Material and Methods. Units for mean and range are centimeter, growing degree days and scores on a 0-10 scale, for PH, HD and St, respectively.

In BP, observational units were plants within half-sib families from a given genotype (maternal parent) *i*. Half-sib families were arranged in a randomized complete block design and assayed in multiple years; so the following mixed model was fitted to phenotypic measurements $P_{ijlm}$, to estimate half-sib family effects *f_i_*’s:

$$P_{ijlm}=\;\mu +f_{i}+r_{j}+{\left(f\times r\right)}_{ij}+t_{l}+{\left(t\times r\right)}_{jl}+{\left(f\times t\right)}_{il}+{\left(f\times t\times r\right)}_{ijl}+\varepsilon_{ijlm}$$

where μ is the population mean; $f_{i}$, $r_{j}$ and $t_{l}$ are the effects of half-sib family i (fixed), block j (random) and year l (random) respectively; × indicates interactions (random); $\varepsilon_{ijlm}$ are residuals for plant *m* within plot *ij* in year *l*. In AP, observational units were clones of a given genotype *i*. Genotypes were arranged in a sets-in-reps design and assayed in multiple years; so the following model was fitted to measurements $P_{ijkl}$ to estimate genotype effects *g_i_*’s:

$$P_{ijkl}=\;\mu +g_{i}+r_{j}+s_{jk}+{\left(g\times r\right)}_{ij}+t_{l}+{\left(t\times r\right)}_{jl}+{\left(t\times s\right)}_{jkl}+{\left(g\times t\right)}_{il}+e_{ijl}$$

where μ is the panel mean; $g_{i}$, $r_{j}$, $s_{jk}$ and $t_{l}$ are the effects of genotype i (fixed), replicate j (random), set *k* within replicate *j* (random) and year l (random) respectively; × indicates interactions (random); $e_{ijl}$ are residuals for clone *ij* in year *l*.

The models described above conform to analyses of strip-plot (split-block) designs (Steel *et al.* 1996), in which years and genotype classes (half-sib families in BP, individual genotypes in AP) are whole-plot factors in cross-classification and sub-plot factors are combinations of years and genotype classes. For each random term, the corresponding effects were modeled as independent and identically normally distributed. The linear mixed models described above were fitted using ASREML-R (Butler *et al.* 2009).

Effects *f_i_*’s in BP are transmitting abilities of genotypes (the mean of their half-sib progeny in their respective breeding population), so $f_{i}=\frac{BV_{i}}{2}$, where *BV_i_* is the breeding value of genotype *i*. In comparison, effects *g_i_*’s in AP are genotypic values, such that $g_{i}=BV_{i}+\Delta_{i}$, where $\Delta_{i}$ is the deviation from additivity due to dominance and/or epistasis. Thus, outcomes of interest for genomic prediction were set to be genotype means *y_i_*’s such that $y_{i}=\hat{\mu }+2{\hat{f}}_{i}$ in BP and $y_{i}=\hat{\mu }+{\hat{g}}_{i}$ in AP, with $\hat{\mu }$ the estimated population mean in BP or estimated panel mean in AP.

### Population structure and relatedness

#### Admixture analysis:

**Figure 1:**
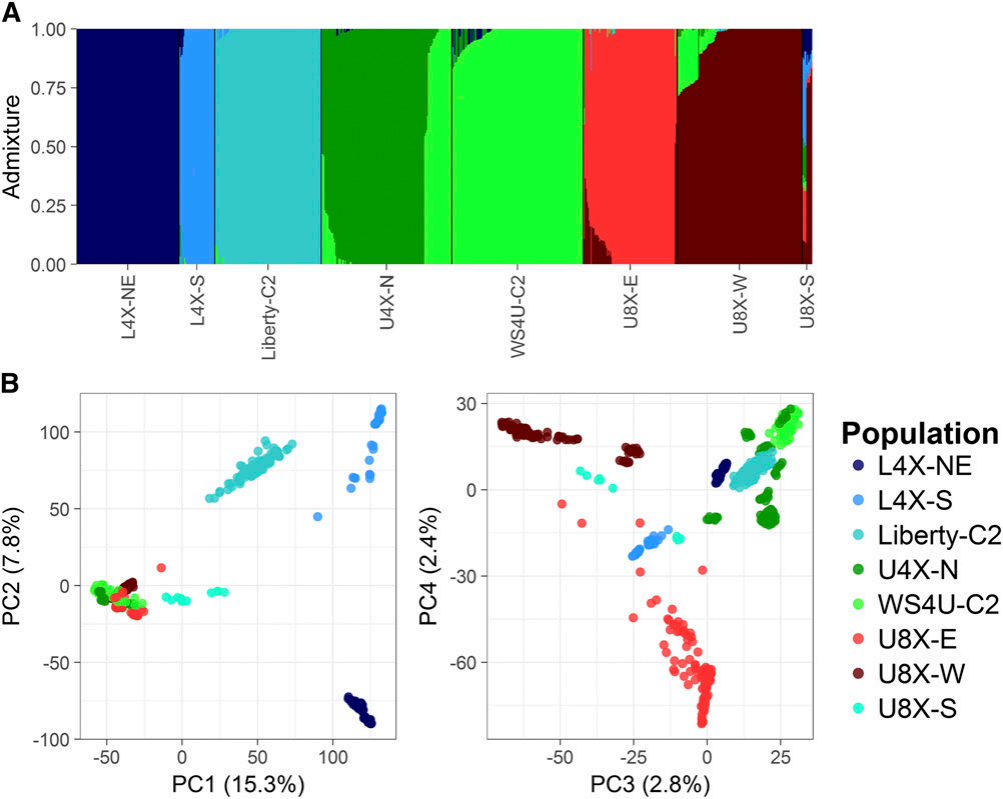
Population structure in the sample (A) Admixture plot of the whole sample, with colors designating the seven inferred population clusters, which roughly matched populations, with the exception of U8X-S which displayed strong admixture; (B) Principal component analysis (PCA) plot of the whole sample of 760 individuals, with colors designating the eight populations.

The soft clustering model from the ADMIXTURE software was fitted on the whole sample and the whole set of SNPs, *i.e.*, without selection on individuals or markers (Alexander *et al.* 2009). Based on the 10-fold cross-validation implemented in ADMIXTURE (Alexander and Lange 2011), the number of population clusters in the admixture model was set to K=7, as cross-validation error reached a plateau at that value (Figure S1). The resulting n×K matrix **A** of admixture coefficients comprised inferred membership probabilities at each cluster (Figure 1a). For convenience (in prediction models), minimum values in **A** (10^−5^) were set to zero while ensuring that each row still summed to one (by dividing each element in **A** by its row sum).

#### Principal component analysis:

Principal component analysis (PCA) was performed on the whole sample and the whole set of SNPs. The number of principal components (PCs) to choose for depicting population structure was chosen based on the proportion of variance explained and the grouping patterns captured by PCs (Figure 1b). The resulting n×d PC matrix **P** consisted of coordinates for each individual at the first d=4 PCs.

#### Recent relationships among individuals:

Let $G=\dot{X}{\dot{X}}^{\prime }/v$ be the genomic relationship matrix as defined by VanRaden (2008), where $\dot{X}$ is the centered marker-data matrix and $v=2\overset{m}{\underset{l=1}{\sum }}{\hat{\pi }}_{l}\left(1-{\hat{\pi }}_{l}\right)$ is a scaling factor depending on allele frequencies ${\hat{\pi }}_{l}$′s (estimated on the whole sample), where m=717,814 is the number of SNP markers. Following Fan *et al.* (2013), **G** was decomposed as $G=PP^{'}+G_{P}$, where P consisted of the first d=4 PCs, as described above. Matrix $PP^{'}$ is the dense part of the relationship matrix **G**, representing resemblance among individuals through population structure, whereas matrix $G_{P}$ represents recent relationships conditional on population structure, similarly to the adjusted relationships introduced by Thornton *et al.* (2012) and Conomos *et al.* (2016), with the difference that here coefficients in $G_{P}$ are not scaled for direct estimation of recent-kinship coefficients. Here the graphical LASSO was applied to $G_{P}$ to infer a graph of recent relationships among individuals, according to a regularization parameter *λ*. Parameter *λ* was chosen to maximize the restricted likelihood in a GBLUP model based on the regularized genomic relationship matrix $\tilde{G}$, fitted to the whole sample (see Appendix 1 for technical details and discussion on graph inference). The GBLUP model depicting relationships through $\tilde{G}$ was fitted for each trait separately, so different optimal values of *λ* were inferred for different traits.

### Genomic prediction models

All linear mixed models described below were fitted using the R package rrBLUP (Endelman 2011).

For a given marker-data matrix **X** and vector **y** of outcomes, the standard GBLUP model is described as follows:

$$y=\text{Q}\alpha +u+e;\left[\begin{array}{c} u\\ e \end{array}\right]\;∼\;N\left(\left[\begin{array}{c} 0\\ 0 \end{array}\right],\left[\begin{array}{cc} XX^{\prime }\sigma_{\beta }^{2} & 0\\ 0 & I_{n}\sigma_{e}^{2} \end{array}\right]\right)$$ (1)

where **y** is the *n*-vector of genotype means (*y_i_*′s, as described above); **X** is the n×m marker-data matrix (here consisting of allelic dosages), and $\sigma_{\beta }^{2}$ is the variance of marker effects; **Q** is the model matrix for fixed effects **α**; $I_{n}\sigma_{e}^{2}$ is the covariance matrix for errors considered independent and identically distributed.

Hereafter, the testing set TS is defined as the set of individuals left out for model validation. The calibration set CS is the set of individuals used to fit the prediction models, which excludes the TS but does not necessarily consist of all remaining (available) individuals.

We defined the mean structure in fitted models by **Q** being one of the following: *(Intercept*) a *n*-vector of ones $1_{n}$, such that fixed effects consisted of a single intercept; (*PCA*) the n×5 matrix $\left[\begin{array}{cc} 1_{n} & P \end{array}\right]$ of column vector of ones and first four PCs; (*Panel*) the n×2 model matrix attributing observations to panel AP or BP, such that fixed effects reflected differences in genetic compositions and environments across panels; or (*Group*) the n×7 matrix model matrix attributing individuals to the following putative population groups: WS4U-C2, Liberty-C2, U4X-N, U8X-W+U8X-S, U8X-E, L4X-NE and L4X-S. Genotypes from U8X-S were grouped with U8X-W on the basis of their proximity according to the first 4 PCs, to avoid having one group with too few observations.

In this study, we first compared mean structures with respect to prediction accuracy under the standard prediction procedure (*GBLUP*, as described below). Then, we focused on $\text{Q}=1_{n}$ and compared prediction procedures for accommodating population heterogeneity (see below; *GBLUP*, *GBLUP-Target*, *MPM-Mixture*, *MPM-Matérn*). Prediction accuracy of models (differing either by mean structure or prediction procedure) was assessed by cross-validation as described in the next subsection (Validations).

#### Whole-sample model: GBLUP:

In the whole-sample model (*GBLUP*), we fitted model (1) to all available individuals, thereby assuming that the whole sample consists of only one population. This method consists of ignoring population heterogeneity and relying on robustness of standard GBLUP to interactions between markers and population backgrounds.

#### Target-population model: GBLUP-Target:

In the target-population model (*GBLUP-Target*), we fitted model (1) to individuals belonging to the same population as the TS, when possible (see below). This method corresponds to a typical choice of reducing population heterogeneity and basing predictions only on individuals that have genetic backgrounds that are *a priori* similar to those in the TS.

#### Multi-population models: MPM-Mixture, MPM-Matérn:

Multi-population models (*MPM*) were extensions of model (1) intended to accommodate population heterogeneity. The following general model was fitted:

$$y=\text{Q}\alpha +u+e;\left[\begin{array}{c} u\\ e \end{array}\right]∼N\left(\left[\begin{array}{c} 0\\ 0 \end{array}\right],\left[\begin{array}{cc} \left(\Omega_{n}○XX^{\prime }\right)\sigma_{\beta }^{2} & 0\\ 0 & I_{n}\sigma_{e}^{2} \end{array}\right]\right)$$ (2)

where ○ is the element-wise (Hadamard) product, and $\Omega_{n}$ is a *n* × *n* covariance matrix depicting population differentiation among individuals (see Appendix 2 for derivations and technical details). To parsimoniously estimate $\Omega_{n}$, we used two different procedures: *MPM-Mixture* (based on **A**) and *MPM-Matérn* (based on **P**). In both procedures, we did not model any heteroscedasticity for additive genetic effects **u**.

In *MPM-Mixture* (the reference *MPM* procedure), $\Omega_{n}=\rho A\Theta_{K}A^{'}+\left(1-\rho \right)J_{n}$, where $J_{n}$ is the *n* × *n* matrix of ones and $\Theta_{K}$ is a *K* × *K* matrix depicting relationships among population clusters as inferred in **A**. Here, we simply set $\Theta_{K}=I_{K}$ ($I_{K}$ is the *K* × *K* identity matrix), so $\Omega_{n}=\rho AA^{'}+\left(1-\rho \right)J_{n}$. Therefore in this procedure, ρ∈[0,1] set a trade-off between the case where relationships were cluster-specific (ρ=1) and the case where relationships assumed one single homogeneous population for all individuals (ρ=0). This approach is similar (but not exactly equivalent) to the *K*-kernel method of Heslot and Jannink (2015), which set a similar balance between cluster-specific and overall relationships, but using G for relationships (VanRaden 2008), instead of $XX^{\prime }$, and considering only discrete population clusters (in which case values in **A** would then be only 0 or 1). Alternatively, *MPM-Mixture* may be viewed as a multi-kernel model where $\rho \sigma_{\beta }^{2}$ and $\left(1-\rho \right)\sigma_{\beta }^{2}$ are the variance components respectively associated to cluster-specific and main marker effects.

In *MPM-Matérn* (the proposed *MPM* procedure),$\;\Omega_{n}={\left(\kappa_{\nu ,h}\left(p_{i},p_{j}\right)\right)}_{n\times n}$, where $\kappa_{\nu ,h}$ is a Matérn kernel function of $p_{i}$ and $p_{j}$: $\kappa_{\nu ,h}\left(p_{i},p_{j}\right)=\frac{2^{1-\nu }}{\Gamma \left(\nu \right)}{\left(\sqrt{2\nu }\frac{{\left\|p_{i}-p_{j}\right\|}_{2}}{h}\right)}^{\nu }R_{\nu }\left\{\sqrt{2\nu }\frac{{\left\|p_{i}-p_{j}\right\|}_{2}}{h}\right\}$, ${\left\|p_{i}-p_{j}\right\|}_{2}$ is the Euclidean distance between the *d*-vectors of PC coordinates for any pair (*i*, *j*) of individuals, ν > 0 is a shape parameter, h > 0 is a scale parameter, and $R_{\nu }\left\{\cdot \right\}$ is the modified Bessel function of the second kind, of order *ν* (Abramowitz and Stegun 1984, Ober *et al.* 2011). Matérn functions have been used in various contexts, including in genomic prediction for depicting relationships among individuals (Ober *et al.* 2011). Here, we used Matérn functions to depict relationships among populations, with the input ${\left\|p_{i}-p_{j}\right\|}_{2}$ representing differentiation with respect to population structure in d=4 orthogonal directions. We used Matérn functions instead of more typical kernel functions (*e.g.*, an exponential or Gaussian kernel function) to allow for some flexibility in the shape of the correlation in $\Omega_{n}$: ν=0.5 and ν=∞ correspond respectively to the exponential and Gaussian kernels as special cases, while different shapes can also be fitted (Ober *et al.* 2011).

The parameter *ρ* in *MPM-Mixture* was estimated by maximizing the restricted likelihood of model (2) using the optimization algorithm implemented in the R function *optimize*. The parameters *ν* and *h* in *MPM-Matérn* were estimated by maximizing the restricted likelihood of model (2) using the Nelder-Mead algorithm implemented in the R function *constrOptim*, with constraints for positivity. In order to control (to some extent) for the possible presence of local maxima in the restricted likelihood surface in *MPM-Matérn*, we used four different starting points $\left(\nu_{0},h_{0}\right)$: $\left(0.5,D_{max}/2\right)$, $\left(0.5,D_{max}\right)$, $\left(10,D_{max}/2\right)$ and $\left(10,D_{max}\right)$, with *D_max_* the maximum distance ${\left\|p_{i}-p_{j}\right\|}_{2}$ observed over pairs of individuals (*i*, *j*). In cross-validation (see next section), parameters *ρ*, *ν* and *h* were estimated in each CS separately.

### Validations

We assessed the accuracy of our prediction procedures by cross-validation (CV) under two schemes: whole-sample calibration, where all individuals except the TS are included in the CS, and cross-population calibration, where the target population (the population to which the TS belongs) is excluded from the CS. The target-population model *GBLUP-Target* was only assayed in whole-sample calibration, since this model could only rely on individuals from the target population for calibration (in *GBLUP-Target*, the CS could only consist of individuals in the target population, which was not possible in cross-population calibration).

For each target population (L4X-NE, U4X-N, Liberty-C2 or WS4U-C2), we used as the TS a random subset of the target sample. The size of the TS was one fifth of the target sample size. All remaining individuals were used as input to the prediction procedures. Such validations were replicated $n_{rep}=20$ times for each target.

Prediction procedures were evaluated for accuracy by $c_{TS}=Cor\left(y_{TS},{\hat{y}}_{TS}\right)$, *i.e.*, the correlation between actual and predicted outcomes in a given TS. To assess the significance of differences in prediction accuracy between two procedures, we performed a *t*-test on $T=\frac{\bar{\delta }}{SD\left(\bar{\delta }\right)}$, where $\bar{\delta }$ is the average of $\delta =z\left(c_{t}\right)-z(c_{0})$; $c_{t}$ ($c_{0}$) is the vector of prediction accuracies over testing sets for the tested procedure (baseline procedure); and z is the Fisher transformation. The standard error of the mean difference in prediction accuracy, $SD\left(\bar{\delta }\right)$, was estimated in two different ways: (liberal *t*-test) $SD\left(\bar{\delta }\right)=SD\left(\delta_{TS}\right)\sqrt{\frac{1}{n_{rep}}}$ where $SD\left(\delta_{TS}\right)$ is the standard deviation of δ, with all testing sets assumed to be independent datasets; (conservative *t*-test) based on the first method of Nadeau and Bengio (2003), $SD\left(\bar{\delta }\right)=SD\left(\delta_{TS}\right)\sqrt{\frac{1}{n_{rep}}+\frac{o}{1-o}}$, where redundancy over testing sets is accounted for by the additional term $\frac{o}{1-o}$, with *o* being the expected fraction of overlap among testing sets; here $o=\frac{1}{5}$ and $\frac{o}{1-o}=\frac{1}{4}$ because testing sets were random subsets consisting of a fifth of any given target sample. We considered that this method for estimating $SD\left(\bar{\delta }\right)$ was conservative in whole-sample calibration because Nadeau and Bengio (2003) derived it by assuming that the CV criterion (the “loss function”, analog here to $z\left(c_{TS,t}\right)-z(c_{TS,0})$, for a given TS) did not depend on the CS instances, given a particular CS size. Therefore the adjustment from Nadeau and Bengio (2003) may have overestimated the correlation among values of the CV criterion across replicates, in whole-sample calibration, since prediction procedures are probably quite sensitive to differences in the composition of the CS. In all comparisons between procedures, we reported the results from both tests in order to characterize the significance of differences in prediction accuracy.

### Data availability

Population information (population assignment and geographical origin of genotypes, when available), raw phenotypic data (trait measurements at individual plants) and estimated genotype means (for maternal parents in BP and individuals in AP) are available in Files S1, S2 and S3, respectively. These supplementary files as well as the marker data (allelic dosages at the 717,814 selected SNP markers; in .rds format readable in R) are available from figShare. Supplemental material available at Figshare: https://doi.org/10.25387/g3.7464863.

## Results

### Population structure in the sample

#### Population-level differentiation:

Seven population clusters were inferred from the ADMIXTURE software (Figure S1; Alexander *et al.* 2009). These clusters corresponded roughly to populations L4X-NE, L4X-S, Liberty-C2 and U4X-N, WS4U-C2, U8X-E, U8X-W. One population with little representation in our sample, U8X-S, appeared to be of mixed origin (Figure 1a). The other populations generally displayed a low level of admixture, with relatively few individuals having intermediate admixture coefficients. There seemed to be some admixture involving upland populations (WS4U-C2 and U4X-N, WS4U-C2 and U8X-W, U8X-E and U8X-W), with even some shared ancestry between WS4U-C2 and U4X-N. The PCA confirmed that population structure was relatively discrete (Figure 1b). Expectedly, the first PC separated genotypes by ecotype while the second PC reflected geographical origin within the lowland ecotype (Lu *et al.* 2013, Evans *et al.* 2015). The third and four PCs discriminated upland genotypes by geographical origin and ploidy level, and distinguished L4X-S from the two other lowland populations (L4X-NE and Liberty-C2).

Differences in mean and range among populations were quite typical of previously reported differences between ecotypes (Table 1; Casler 2012). Indeed, L4X-S and Liberty-C2 (populations of lowland origin) had high mean values and range values for PH, HD and St, compared to upland populations (excluding U8X-S). However, L4X-NE stood out as a lowland population for being relatively short, early-flowering, and prone to lodging, with corresponding values for PH, HD, and St more similar to those of the upland populations.

#### Recent relationships in the sample:

**Figure 2:**
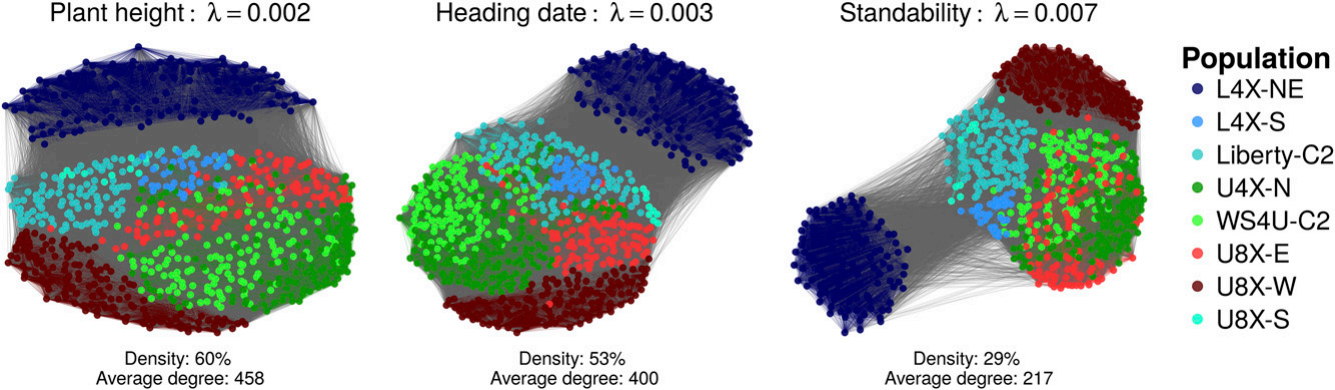
Inferred graphs of relationships, conditional on population structure Each graph represents the relationships as depicted by the graphical LASSO applied to the whole sample of individuals. The parameter *λ* represents the degree of regularization on conditional relationships, fitted by maximum restricted likelihood for each trait, in a GBLUP model based on regularized relationships (Appendix 1). Nodes (individuals) were positioned using the force-directed placement algorithm of Fruchterman and Reingold (1991), as implemented in function ggnet (R package GGally), so aggregation of nodes reflects connectedness.

Here, marginal genomic relationships were defined as the elements of $G=\dot{X}{\dot{X}}^{\prime }/\nu$, with $\dot{X}$ consisting of centered marker variables, and *ν* being some scaling factor. The strong and quite discrete population structure in the sample translated into multimodal marginal genomic relationship coefficients, with the multiple peaks in off-diagonal elements of **G** reflecting differentiation of population with respect to allele frequencies (Figure S2a). Conditioning relationships on population structure (as depicted by the first four PCs of $\dot{X}$) yielded the matrix $G_{P}$, with $G_{P}=G-PP^{'}$ and **P** reflecting structure in **G** due to population-level variation (Fan *et al.* 2013). The conditional genomic relationships seemed sparser, in the sense that they appeared to cluster around zero, so most individuals could be assumed to be unrelated after accounting for population structure in the sample (Figure S2b). Conditional relationships in $G_{P}$ were particularly relevant in this study, since among-population variation, captured by $PP^{'}$, contributed little to variation within any given TS. Indeed, any TS generally consisted of selection candidates from a relatively homogeneous target sample (made of individuals from WS4U-C2, Liberty-C2, U4X-N or L4X-NE), where variation with respect to P was minimal. Graphs of recent relationships, inferred by the graphical LASSO, were rather dense, with average degrees (number of neighbors by node/individual in the graph) ranging from 217 to 458 (Figure 2). However, some noticeable features of populations emerged from the inferred graphs (Figure 2): WS4U-C2, U4X-N and U8X-E appeared quite connected to one another; U8X-W also showed some connection with other upland populations but seemed more distinct, as reflected by a relatively lower average degree (Figure S3); Liberty-C2 and L4X-S were somewhat connected to both upland and lowland populations, which certainly explains why their individual degrees were generally high (Figure S3); most notably, L4X-NE displayed an outstandingly low level of connection with the other populations, which translated in a clear separation of this population in the graph, after placing the nodes based on a force-directed algorithm (Fruchterman and Reingold 1991). These features exemplify the usefulness of conditional relationships and their associated graphs for describing relationships among individuals.

### Impact of mean structure on prediction accuracy

Prior to assessing different prediction procedures accommodating population heterogeneity, models were compared for different fixed-effect specifications used to characterize population structure (mean structures of models). Mean structures were tested for prediction accuracy under *GBLUP*, in which the whole sample, excluding the TS, was used to fit a standard GBLUP model. In whole-sample calibration (where the target population was included in the CS), the various mean structures assayed differed marginally with respect to their prediction accuracy. There were improvements over *Intercept* (only intercept as fixed effect) by mean structures which explicitly captured population structure, *i.e.*, *PCA* (intercept and effects of PCs), *Panel* (effects of panels AP and BP) and *Group* (effects of putative population groups). However, those were small, inconsistent and moderately significant (hereafter “moderately significant” refers to P≤0.05 based on the liberal “naïve” *t*-test) (Table 2a). Conversely, in cross-population calibration (where the target population was excluded from the CS), fixed effects explicitly depicting population structure resulted in highly significant decreases in prediction accuracy compared to *Intercept* (hereafter “highly significant” refers to P≤0.05 based on the conservative *t*-test adapted from Nadeau and Bengio 2003; see *Material and methods* for details). In particular, prediction accuracy was substantially lower with *PCA* for L4X-NE (HD, St), as well as with *Group* for U4X-N (PH, HD), L4X-NE (PH, HD) and Liberty-C2. Mean structure *Panel* was not as sensitive to cross-population calibration compared to *PCA* and *Group*, and even showed one highly significant increase in prediction accuracy compared to *Intercept*, for Liberty-C2 (PH). But it also showed decreases in prediction accuracy, with U4X-N (PH) and L4X-NE (HD), which were stronger and highly significant (Table 2b). Interestingly, the deterioration of prediction accuracy in cross-population calibration by *PCA* and *Group* may be due to different factors. Indeed, while *PCA* may fail to properly extrapolate effects of PCs outside the set of populations represented in the CS, *Group* would fail to capture any difference due to population differentiation in the TS (since all individuals in the TS belong to the same unobserved population group). Due to the relative stability in performance of *Intercept*, hereafter we chose to focus on this mean structure when comparing prediction procedures.

**Table 2:** Average prediction accuracy by mean structure

		a) Target included in CS				b) Target population omitted from CS			
Trait	Population	*Intercept*	*PCA*	*Panel*	*Group*	*Intercept*	*PCA*	*Panel*	*Group*
**PH**	WS4U-C2	0.163 (0.121)	0.163 (0.121)	0.164 (0.123)	0.180 (0.122)	0.230 (0.144)	0.234 (0.145)	0.217 (0.146) •	0.225 (0.142)
	Liberty-C2	0.476 (0.189)	0.477 (0.189)	0.478 (0.189)	0.469 (0.191)	0.025 (0.208)	0.030 (0.202)	0.045 (0.209) *	−0.048 (0.196) •
	U4X-N	0.526 (0.149)	0.527 (0.147)	0.526 (0.143)	0.525 (0.136)	0.271 (0.162)	0.258 (0.166) •	0.247 (0.164) *	0.067 (0.175) *
	L4X-NE	0.767 (0.074)	0.766 (0.074) •	0.766 (0.074) •	0.771 (0.072) •	0.403 (0.179)	0.391 (0.184) *	0.405 (0.180)	0.355 (0.189) *
**HD**	WS4U-C2	0.272 (0.185)	0.276 (0.186) •	0.291 (0.177) •	0.282 (0.173)	0.122 (0.166)	0.132 (0.167) •	0.129 (0.140)	0.140 (0.140)
	Liberty-C2	0.532 (0.145)	0.538 (0.141) •	0.536 (0.146) •	0.516 (0.158)	0.125 (0.185)	0.142 (0.177)	0.127 (0.185)	0.080 (0.204) •
	U4X-N	0.694 (0.103)	0.693 (0.103)	0.689 (0.114)	0.694 (0.110)	0.447 (0.179)	0.438 (0.163)	0.406 (0.191) •	0.388 (0.167) •
	L4X-NE	0.828 (0.074)	0.826 (0.074) *	0.828 (0.074) •	0.832 (0.073) •	0.401 (0.212)	0.338 (0.222) *	0.377 (0.222) *	0.229 (0.206) *
**St**	WS4U-C2	0.070 (0.208)	0.074 (0.209)	0.079 (0.205) •	0.078 (0.208)	−0.046 (0.193)	-0.028 (0.195) *	−0.047 (0.191)	−0.051 (0.192)
	Liberty-C2	0.116 (0.248)	0.110 (0.248)	0.114 (0.248)	0.098 (0.250) *	0.164 (0.185)	0.145 (0.231)	0.153 (0.177)	0.104 (0.150) •
	U4X-N	0.265 (0.169)	0.264 (0.167)	0.270 (0.172)	0.281 (0.171) •	0.048 (0.218)	−0.067 (0.209) •	−0.000 (0.219) •	0.020 (0.207)
	L4X-NE	0.589 (0.127)	0.588 (0.128)	0.589 (0.126)	0.590 (0.126)	0.090 (0.219)	−0.330 (0.172) *	0.096 (0.218) •	0.103 (0.227) •

In parentheses: standard deviation across cross-validation replicates. Validation scheme: (a) whole-sample calibration, where individuals in the target population, except the selection candidates, are included in the calibration set; (b) cross-population calibration, where all individuals from the target population are omitted from the calibration set. Trait: plant height (PH), heading date (HD) or standability (St). Population: population used as target for prediction. Prediction accuracies are averaged over 20 cross-validation replicates. Models differ by mean structure (fixed-effect specification), under the same prediction procedure (*GBLUP*: whole-sample model). *Intercept*: only an intercept; *PCA*: intercept and effects of first four PCs; *Panel*: effect of panels (AP, association panel; BP, breeding panel); *Group*: effects of putative population groups (WS4U-C2, Liberty-C2, U4X-N, U8X-W+U8X-S, U8X-E, L4X-NE and L4X-S). Comparisons to *Intercept*: •: *P* ≤ 0.05 in unadjusted (naïve) *t*-test (liberal); *: *P* ≤ 0.05 in *t*-test corrected for overlap in testing sets as in Nadeau and Bengio (2003) (conservative). Underlined values correspond to the highest prediction accuracy for each validation scheme, trait and population.

### Impact of prediction procedures on prediction accuracy

#### Target-population model:

For prediction in a given TS, the target-population model (*GBLUP-Target*) consisted in restricting the CS to the subset of the sample belonging to the same population as the TS. Compared to *GBLUP*, the target-population model yielded decreases in prediction accuracy which appeared moderately significant for PH (WS4U-C2, U4X-N) and St (Liberty-C2) (Table 3a, Table S1). However, prediction accuracy for St (WS4U-C2) was higher, with a moderately significant difference. More intriguing is the consistent increase in prediction accuracy with L4X-NE, with differences being small yet highly significant for PH and HD, and moderately significant for St. It is unclear whether these differences are due to the consistently higher accuracies achieved with L4X-NE (in *GBLUP-Target*) compared to other populations, or a result of L4X-NE being relatively under-connected to the other populations in the sample (Figure 2, Figure S3). Both factors could very well contribute to the observed decreases in accuracy when incorporating information from the whole sample.

**Table 3:** Average prediction accuracy by prediction procedure

		a) Target included in CS				b) Target population omitted from CS		
Trait	Population	*GBLUP*	*GBLUP-Target*	*MPM-Mixture*	*MPM-Matérn*	*GBLUP*	*MPM-Mixture*	*MPM-Matérn*
**PH**	WS4U-C2	0.163 (0.121)	0.115 (0.123) •	0.163 (0.121)	0.133 (0.124)	0.230 (0.144)	0.213 (0.137) •	−0.074 (0.214) *
	Liberty-C2	0.476 (0.189)	0.467 (0.186)	0.476 (0.189)	0.470 (0.186)	0.025 (0.208)	0.025 (0.209)	0.122 (0.216) •
	U4X-N	0.526 (0.149)	0.486 (0.160) •	0.525 (0.149)	0.540 (0.130)	0.271 (0.162)	0.253 (0.160) •	0.265 (0.168)
	L4X-NE	0.767 (0.074)	0.782 (0.068) *	0.767 (0.074) •	0.762 (0.076) •	0.403 (0.179)	0.403 (0.179)	0.153 (0.188) *
**HD**	WS4U-C2	0.272 (0.185)	0.273 (0.159)	0.254 (0.178)	0.332 (0.145)	0.122 (0.166)	0.094 (0.150)	0.269 (0.151) •
	Liberty-C2	0.532 (0.145)	0.533 (0.152)	0.516 (0.152)	0.524 (0.153)	0.125 (0.185)	0.137 (0.191)	0.171 (0.181)
	U4X-N	0.694 (0.103)	0.693 (0.110)	0.703 (0.100) *	0.724 (0.090) •	0.447 (0.179)	0.297 (0.163) *	0.514 (0.136) •
	L4X-NE	0.828 (0.074)	0.841 (0.072) *	0.832 (0.073) *	0.835 (0.068)	0.400 (0.212)	0.352 (0.212) *	0.527 (0.177) *
**St**	WS4U-C2	0.070 (0.208)	0.115 (0.187) •	0.067 (0.213)	0.075 (0.198)	−0.046 (0.193)	−0.015 (0.201) •	0.031 (0.177) •
	Liberty-C2	0.116 (0.248)	0.055 (0.234) •	0.105 (0.251) •	0.102 (0.252)	0.164 (0.185)	0.161 (0.190)	0.172 (0.224)
	U4X-N	0.265 (0.169)	0.255 (0.174)	0.269 (0.172)	0.266 (0.166)	0.048 (0.218)	0.042 (0.211)	−0.040 (0.204) •
	L4X-NE	0.589 (0.127)	0.604 (0.120) •	0.590 (0.127)	0.591 (0.129)	0.090 (0.219)	0.122 (0.219) •	0.174 (0.203) *

In parentheses: standard deviation across cross-validation replicates. Validation scheme: (a) whole-sample calibration, where individuals in the target population, except the selection candidates, are included in the calibration set; (b) cross-population calibration, where all individuals from the target population are omitted from the calibration set. Trait: plant height (PH), heading date (HD) or standability (St). Population: population used as target for prediction. Prediction accuracies are averaged over 20 cross-validation replicates. Models differ by prediction procedure, under the same mean structure (*Intercept*: intercept-only model). *GBLUP*: whole-sample model; *GBLUP-Target*: GBLUP model where the CS includes only the individuals from the same population as the TS; *MPM*: multi-population model with among-population correlations based on admixture coefficients (*MPM-Mixture*) or PC distances (*MPM-Matérn*). Comparisons to *GBLUP*: •: *P* ≤ 0.05 in unadjusted (naïve) *t*-test (liberal); *: *P* ≤ 0.05 in *t*-test corrected for overlap in testing sets as in Nadeau and Bengio (2003) (conservative). Underlined values correspond to the highest prediction accuracy for each validation scheme, trait and population.

#### Multi-population models and marker-by-population interactions:

**Figure 3:**
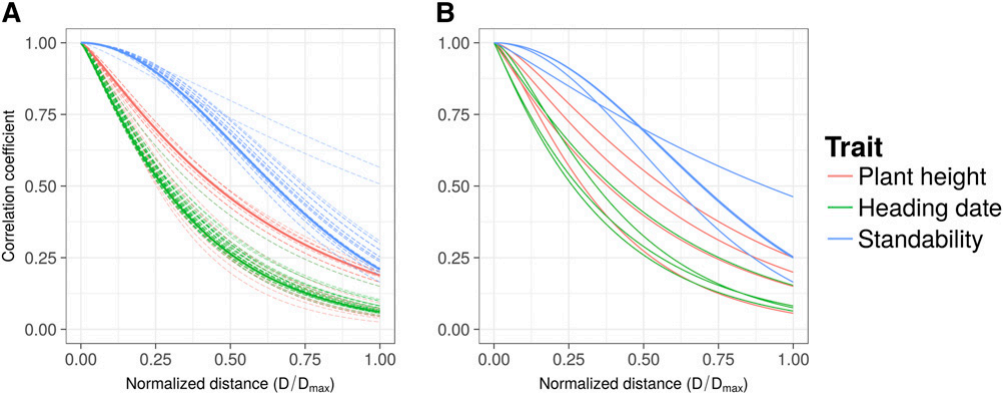
Shape of the inferred correlation functions in *MPM-Matérn* Validation scheme: (A) whole-sample calibration, where individuals in the target population (except the selection candidates) are included in the calibration set; (B) cross-population calibration, where all individuals from the target population are omitted from the calibration set. In (A), dashed curves depict correlation functions inferred in cross-validation replicates (where a part of the target population is included in the testing set), while solid curves depict correlation functions inferred in the whole sample. In (B), solid curves depict correlation functions inferred while omitting one of the four target populations in the calibration set. Correlations are functions of $D/D_{max}$, where *D* is the Euclidean distance between population-structure PCs for any pair of individual (*i*,*j*) and *D_max_* is the maximum of *D* over the whole sample.

The inferred mixing parameter *ρ* from the *MPM-Mixture* model was null (or close to null), low and intermediate, for PH, St and HD respectively, with estimations being quite consistent over CV replicates (Table 4). The improvement in fit, relatively to *GBLUP*, was non-significant for PH, rather significant (*P* < 0.05) for St, and strongly significant (*P*  <  0.001) for HD (Table 4). In *MPM-Matérn*, the inferred correlation functions differed substantially across traits (Table 4), while being quite consistent over CV replicates in whole-sample calibration and across validation schemes, with similar shapes of the correlation function $\kappa_{\nu ,h}$ in whole-sample calibration and cross-population calibration (Figure 3): $\kappa_{\nu ,h}$ roughly resembled an exponential kernel with PH and HD, and was more similar to a Gaussian kernel with St, for which a “shoulder” maintained high correlation in marker effects for individuals that were relatively close to each other, based on their PCs. Remarkably, the shapes of inferred correlation functions were quite consistent in cross-population calibration, despite entire populations being left out from one CS to another (Figure 3b). Inferences regarding among-population correlations ($\Omega_{n}$) in *MPM-Matérn* were weakly significant for PH and St, with *p*-values close to 0.05; in contrast, inferences regarding $\Omega_{n}$ for HD were strongly significant, with *P* < 0.001 (Table 4). Interestingly, distances based on PCs may be equivalent to distances based on allele frequencies. Specifically, ${\left\|p_{i}-p_{j}\right\|}_{2}=2{\left\|\pi_{P}{}_{i}-\pi_{P}{}_{j}\right\|}_{2}$, where $\pi_{P}{}_{i}$ ($\pi_{P}{}_{j}$) is the *m*-vector of individual-specific allele frequencies of individual *i* (*j*) as described by Conomos *et al.* (2016), with population structure described by $\left[\begin{array}{cc} 1_{n} & P \end{array}\right]$ (Appendix 3). Therefore, the significant relationship between PC-based distances and correlations in marker effects (depicted by $\Omega_{n}$) for HD in *MPM-Matérn* indicates that marker effects for this trait were highly sensitive to variation in allele frequencies across genetic backgrounds.

**Table 4:** Multi-population model fit: parameter estimates, likelihood-ratio test statistic and *p*-value, by trait and procedure

Trait	Procedure	Parameter estimate	LRT statistic	LRT *p*-value
**PH**	*MPM-Mixture*	*ρ*: 0.000 (0.000-0.060)	0.00 (0.00-0.31)	1.0 (0.58-1.00)
	*MPM-Matérn*	h*: 0.525 (0.287-0.525)	7.39 (2.44-10.66)	0.025 (0.0049-0.29)
		*ν*: 0.625 (0.550-0.825)
**HD**	*MPM-Mixture*	*ρ*: 0.434 (0.299-0.550)	15.89 (9.22-20.15)	6.7×10^−5^ (7.2×10^−6^-0.0024)
	*MPM-Matérn*	h*: 0.325 (0.298-0.488)	42.76 (28.57-40.25)	5.2×10^−10^ (1.8×10^−9^-6.2×10^−7^)
		*ν*: 0.619 (0.520-0.886)
**St**	*MPM-Mixture*	*ρ*: 0.138 (0.136-0.180)	5.72 (5.21-7.99)	0.017 (0.0047-0.023)
	*MPM-Matérn*	h*: 0.134 (0.125-1.100)	7.59 (7.72-9.94)	0.022 (0.0069-0.021)
		*ν*: 9.049 (0.600-10.014)

In parentheses: range of values for every one of the four target populations omitted, in cross-population calibration. Trait: plant height (PH), heading date (HD) or standability (St). *MPM*: multi-population model with among-population correlations based on admixture coefficients (*MPM-Mixture*; *ρ*: mixture parameter) or PC distances (*MPM-Matérn*; *ν*: shape parameter; $h^{*}=h/D_{max}$, with *h* the scale parameter and *D_max_* the maximum distance observed over pairs of individuals). LRT (likelihood-ratio test) statistic: $-2\text{log}\left(L_{0}/L_{1}\right)$ where *L_0_* and *L_1_* are the restricted maximum likelihoods of *GBLUP* and one of the *MPM* models, respectively; *p*-values were obtained from a *χ^2^*-ditribution with one (*MPM-Mixture*) or two (*MPM-Matérn*) degrees of freedom.

In whole-sample calibration, the performance of *MPM-Mixture* was very similar to that of *GBLUP*, with differences in accuracy ranging from -0.018 to +0.009 (Table 3a). Quite surprisingly, *MPM-Mixture* displayed slightly deteriorated accuracies for HD (with the exception of U4X-N), despite the strongly significant improvement in fit for this trait. In contrast, *MPM-Matérn* yielded larger differences in accuracy, ranging from -0.019 to +0.060 in whole-sample calibration (Table 3a). With the two upland target populations (WS4U-C2 and U4X-N), noteworthy increases in prediction accuracy (+0.060 and +0.030 respectively) were observed for HD. But with the two other target populations (Liberty-C2 and L4X-NE), smaller differences in accuracy (-0.008 and +0.007 respectively) were observed for HD.

In cross-population calibration, *MPM-Mixture* showed more differences in accuracy compared to *GBLUP*, with differences in accuracy ranging from -0.150 to 0.032 (Table 3b). Again, *MPM-Mixture* displayed deteriorated accuracy for HD despite a strong improvement in fit, with a dramatic decrease by 0.15 for U4X-N. Such decrease in accuracy may be due to the lack of flexibility of *MPM-Mixture* in depicting among-population resemblance, as it only fits one correlation coefficient for all pairs of populations. In cross-population calibration, *MPM-Matérn* also resulted in large differences in accuracy compared to *GBLUP*, ranging from -0.304 to 0.147. The dramatic decreases in prediction accuracy with PH (-0.304 and -0.250 for WS4U-C2 and L4X-NE, respectively) could be explained by the relatively weak improvement in model fit from *GBLUP* to *MPM-Matérn* (Table 4). Interestingly, large and significant improvements in prediction accuracy were observed with HD, similarly to the results from whole-sample calibration, with nonetheless more dramatic increases in accuracy. While *MPM-Mixture* simply estimates a general coefficient for among-population resemblance, based on the CS, *MPM-Matérn* may be more suitable for extrapolation to unobserved population backgrounds, as it estimates the resemblance between any two populations as a function of their specific properties (here, PC coordinates). Consistently, the relative improvement in accuracy from *GBLUP* to *MPM-Matérn* seemed more predictable based on the relative improvement in model fit. Specifically, a decrease in Bayesian information criterion (BIC) seemed to discriminate cases where an improvement in accuracy could be achieved by *MPM-Matérn*, especially in cross-population calibration where a correct depiction of population heterogeneity seemed more critical (Figure S4).

## Discussion

### Conclusions

The present study assessed different mean structures to represent population differentiation and evaluated various procedures to accommodate population heterogeneity in diverse samples, with an application in switchgrass. We considered different approaches to reflect population structure, *i.e.*, characterizing it implicitly by random marker effects, using only an intercept as fixed effect (*Intercept*), or characterize it explicitly, by continuous differentiation (*PCA*) or discrete effects at the level of panels (*Panel*) or putative population groups (*Group*). Furthermore, we employed three typical strategies for dealing with marker-by-population interactions, *i.e.*, ignoring (*GBLUP*), reducing (*GBLUP-Target*), or modeling (*MPM*) the source of heterogeneity in the data.

Our assessment of mean structures points to a simple fixed-effect specification being preferable in genomic prediction analyses, since accuracies from *Intercept* were relatively high across populations and traits, and relatively stable across validation schemes (whole-sample calibration or cross-population calibration). These conclusions are consistent with those of Phocas and Laloë (2004), who showed larger prediction errors in cattle when including putative genetic groups as fixed effects (comparable to *Group* and *Panel*). Notably, deteriorations of prediction accuracies from *PCA* and *Group* were especially large in cross-population calibration in which entire populations were excluded from the CS. Moreover, these were often noted for L4X-NE which was under-connected to other populations in the sample (Figure 2). Decreases in accuracy with *PCA* suggest that linear fixed effects capturing population structure may fail to properly extrapolate on unobserved populations whose genetics may differ markedly from other populations in the sample (Figure 1b). However, it is worth noting that the switchgrass sample under study was highly structured. Samples in other species, *e.g.*, in maize or rice, may not display such discrete population differentiation and therefore may not suffer as much from fixed effects at population level (Guo *et al.* 2014).

In whole-sample calibration, *GBLUP* often seemed robust to population heterogeneity, regarding prediction accuracy (Table 3a). This robustness was certainly due to the ability of GBLUP models to combine information from individuals according to the specified relationship matrix, by transferring information preferentially from the more related individuals (Searle *et al.* 2009, Habier *et al.* 2013). Furthermore, GBLUP models were probably all the more robust as marker density was high, such that genomic relationships were accurately estimated (Casella and Berger 2002, Endelman and Jannink 2012). However, some decreases in prediction accuracy compared to *GBLUP-Target* suggest that robustness of *GBLUP* may have been affected by other factors. Such factors may be related to relationships within the sample, *i.e.*, under-connectedness of some populations with others (Figure 2), or differences in accuracy of the prediction model across populations, as reflected by *GBLUP-Target* being more accurate in certain populations (Table 3a).

In whole-sample calibration, prediction was mostly determined by individuals in the CS belonging to the same population as the TS. Consistently, *MPM* procedures, which shrink relationships involving individuals from distantly-related populations, did not dramatically affect prediction accuracy (Table 3a). However, in cross-population calibration, decreasing the contribution of individuals from distantly-related populations must have been more pertinent, so that there were more opportunities for improvement of prediction accuracy by *MPM* procedures. In this context, *MPM-Matérn* proved more useful than *MPM-Mixture*, especially with HD for which *MPM-Matérn* resulted in a dramatic improvement of fit (Table 3b). Importantly, this relative superiority may be due to the fact that *MPM-Matérn* extrapolated correlations between populations, through leading PCs, while *MPM-Mixture* merely interpolated such correlations, by estimating a common coefficient of correlation across populations. This lack of flexibility, and the subsequent inability to extrapolate to unobserved populations, must have resulted in high sensitivity of *MPM-Mixture* to the composition of the CS, making it particularly inappropriate in a cross-population context.

Marker-by-population interactions captured by *MPM-Mixture* and *MPM-Matérn* were presumably not confounded by marker-by-environment interactions, since interactions between panel and markers were not significant (*P* > 0.25 in a model, similar to *MPM-Mixture*, which depicted correlation in marker effects between BP, assayed in WI, and AP, assayed in NY; Figure S5). Therefore, models analyzed in this study would reflect actual differences in genetic bases across populations. Moreover, for every trait, genomic variability (variance of marker effects) would be similar across panels. Indeed, the non-significant improvement in fit from an extension of a GBLUP model where genomic variance can vary by panel (*P* ≥ 0.18), suggested limited differences in variance of marker effects by panel (Figure S6). This result further implied that estimation of genotype effects and half-sib family effects (in AP and BP, respectively) and scaling of half-sib family effects (multiplied by two, so they corresponded to breeding values) were effective to ensure concordance in genomic variability across panels.

Here, we modeled interactions between markers and population structure through products of relationships at markers, which were linear ($XX^{\prime }$), and relationships about population structure, which were linear in *MPM-Mixture* and nonlinear in *MPM-Matérn* ($\Omega_{n}$; Appendix 3). Using kernel functions to estimate relationships dispensed us from fitting effects of many variables, by estimating instead *n* breeding values directly from relationships. A similar strategy was adopted quite recently by Jarquín *et al.* (2014), who modeled genotype-by-environment interactions for genomic prediction, through products of linear kernels at markers and linear kernels at environmental covariates. As noted by these authors, such decomposition with respect to interactions had been introduced in quantitative genetics much earlier, by Kempthorne (1954) and Cockerham (1954) for depicting epistatic effects, based on expected relationships under an infinitesimal model. Importantly, relationships about population structure not only allowed us to efficiently specify a genome-wide marker-by-population interaction model, but they also enabled the use of nonlinear kernels at the population level (with $\Omega_{n}$ produced by nonlinear functions in *MPM-Matérn*). Matérn kernels, introduced to genomic prediction research by Ober *et al.* (2011), were used here to estimate covariance among individuals in a flexible yet parsimonious way (Figure 3), while still using simple linear kernels for depicting within-population variability (by $XX^{\prime }$). Our results exemplify the potential usefulness of parsimonious multi-population models, which are all the more interesting that they can be applied on samples comprising many populations. In contrast, typical multi-trait models would be computationally intractable or statistically inefficient here, since those would rely on one parameter for each population pair to model correlations among populations in $\Omega_{n}$ (*e.g.*, 21 parameters for K=7 population clusters). As a matter of fact, multivariate genomic BLUP models fitted by ASREML-R to estimate such correlations among putative population groups (WS4U-C2, Liberty-C2, U4X-N, U8X-W+U8X-S, U8X-E, L4X-NE and L4X-S) failed to converge.

### Improvement of procedures

Our results suggest that a very high increase in quality of fit, as was observed for HD with *MPM-Matérn*, may allow for an increase in accuracy, especially in a cross-population context. In the analysis of Heslot and Jannink (2015) across various multi-population contexts, there seemed to be a positive relationship between differences in quality of fit, as measured by the Akaike information criterion (AIC), and differences in prediction accuracy. Although this relationship was quite loose, it could be noted that for very high increases in AIC (≥30), gains in accuracy were null to high, similarly to the situation of *MPM-Matérn* with HD, for which increases in AIC varied from 28.68 to 42.88, across CV replicates in whole-sample calibration, and from 24.57 to 36.25 in cross-population calibration. Therefore, stringent thresholds on AIC increases could probably be used in *MPM* to avoid relative decreases in accuracy. In this study, one criterion more conservative than the AIC, the BIC, could discriminate cases where prediction accuracy was improved by *MPM-Matérn*, compared to *GBLUP* (Figure S4). Therefore, a possible improvement of *MPM* procedures could simply come from model selection as an integral part of the fitting process, based for example on the BIC. The BIC differences relative to *GBLUP* were almost always negative for PH and St in *MPM*. For these two traits, differences in prediction accuracy from *GBLUP* to *MPM* were quite inconsistent, especially with *MPM-Matérn*, so model selection could probably have made *MPM* procedures more robust. However, such conclusions are based on a restricted set of populations and genetic architectures. So future studies on other datasets would certainly be necessary to test this *post hoc* hypothesis and determine whether criteria such as the BIC can indicate cases where *MPM-Matérn* should be used instead of *GBLUP*.

Another way of potentially improving *MPM* procedures would be to use other types of kernels than those used here. For example, one may use linear kernels based on population-level covariates (*e.g.*, PCs) in place of $AA^{'}$ in *MPM-Mixture*, hence taking an approach similar to that of Jarquín *et al.* (2014). Besides, modeling resemblance among population clusters in *MPM-Mixture*, by $A\Theta_{K}A^{'}$ in place of $AA^{'}$ (where $\Theta_{K}$ captures similarity based on metrics at the population level), could be useful to increase quality of fit, and possibly prediction accuracy. Finally, an interesting way of extending the *MPM* procedures described here would be to incorporate more information at the population level. Here in *MPM*, population homogeneity was captured through admixture coefficients (*MPM-Mixture*) or differences in PC coordinates (*MPM-Matérn*), the latter reflecting differences in allele frequencies (Appendix 3). However, marker-by-population interactions may also be due to differences in LD patterns (Wientjes *et al.* 2016). Therefore metrics depicting such differences could be particularly appropriate for capturing population heterogeneity. Further research would be necessary to determine the type of metrics to use for reflecting differences in LD patterns, and the appropriate way to parsimoniously combine the different types of information regarding population differentiation in *MPM*. Interestingly, geographical distance may succinctly depict population differentiation, due to differences in allele frequencies and/or differences in LD patterns. Fitting population-level correlations in $\Omega_{n}$ as a function of distance of origin would then be particularly useful in species under strong geographical structure, which include switchgrass (Grabowski *et al.* 2014), but also human (Coop *et al.* 2009), as was clearly shown in samples from Europe (Novembre *et al.* 2008), Africa (Bryc *et al.* 2010) and Latin America (Ruiz-Linares *et al.* 2014). Models such as *MPM-Matérn*, which are parsimonious yet flexible in the shape of the fitted correlation function (Figure 3), are promising in various applications on diverse samples, in prediction studies, but also in inferential studies aiming at characterizing the basis for population differentiation.

In this study, marker data were based on exome capture sequencing, which targets a selected subset of exons for sequencing and subsequent SNP calling (Hirsch *et al.* 2014, Evans *et al.* 2014). The potential lack of representation of causal variants by our assay may have resulted in loss of prediction accuracy. While total lack of representation of some genomic regions imposes a limit on prediction accuracy achievable by our procedures, the relative overrepresentation of some genomic regions could be, to some extent, alleviated by genomic relationships which account for correlation among markers and differential degrees of tagging of loci in the marker data (Speed *et al.* 2012, Ramstein *et al.* 2016, Wang *et al.* 2017).

Another limitation in our study is the assumed homogeneity of genetic and residual variances across populations. Here we focused on parsimonious models estimating genetic correlations (not covariances) between populations. Extending *MPM* models to capture variance heterogeneity across populations and/or environments would certainly deserve further investigation. Such models ought to fit functions of variance over genetic and/or environmental variables, similarly to Ou *et al.* (2015) who reported improvements in fit and marginal gains in prediction accuracy in swine, by modeling residual variance over sexes and slaughter dates. Indeed, a decisive advantage of models like *MPM-Matérn* (for correlations) and those of Ou *et al.* (2015) (for variances) is their ability to extrapolate population covariances to genotypes from unobserved population backgrounds.

### Applications and prospects

Based on our case study, we would recommend using *MPM* whenever a strong improvement in model fit is achieved. Otherwise *GBLUP* would be the method of choice, since it is often robust enough to perform at least as well as *GBLUP-Target*. However, fitting a GBLUP model to a CS restricted to the target population may be preferred when making predictions on “outlier populations” such as L4X-NE, which are under-connected to other populations and are characterized by relatively high prediction accuracy in a single-population context. Nevertheless, more empirical studies on population heterogeneity should follow to support the conclusions from our specific application. Such studies could apply to various contexts: in particular, predictions on diverse samples and dynamic breeding programs. The former includes analyses similar to our case study as well as analyses on more complex data, such as historical datasets, in which not only population heterogeneity but also genotype-by-environment interactions must be taken into account (Dawson *et al.* 2013, Rutkoski *et al.* 2015). The latter involves selection across multiple breeding generations, which might not necessarily suffer from strong population heterogeneity (Sallam *et al.* 2015, Auinger *et al.* 2016) but could nonetheless benefit from robust multi-population models for potential increase in persistency of accuracy over generations (Habier *et al.* 2007). In the context of diverse samples or dynamic breeding, simulation studies could also be useful for assessing the suitability of procedures to accommodate population heterogeneity. Differences in allele frequencies and differential LD patterns could be simulated by various genealogies, as was done for example by de Roos *et al.* (2009). Additionally, dependency of marker effects on allele frequencies could be simulated through underlying non-additive genetic effects, as well as allele fixation in specific populations. Indeed, marginal additive marker effects, as captured by linear models such as standard GBLUP, depend on allele frequencies at the loci with which they interact (Hill *et al.* 2008, Mäki-Tanila and Hill 2014, Hill and Mäki-Tanila 2015). Therefore, dominance and epistatic effects could be simulated to generate dependency of marker effects on allele frequencies, which may then be captured by methods such as *MPM-Matérn* (Appendix 3). Though investigations based on simulations would be complex and, to some extent, arbitrary by their choice of genealogies and genetic architectures, they would provide useful frameworks for assessing procedures, such as those presented in this study, in contexts of population heterogeneity.

## Appendix 1

### Inference of recent relationships by the graphical LASSO

Let ${\tilde{G}}_{P}$ be a regularized form of $G_{P}=G-PP^{'}$, with G the genomic relationship matrix as defined by VanRaden (2008), and **P** the matrix of d=4 PCs. We applied the graphical LASSO to $G_{P}$ to infer a sparse matrix ${\tilde{G}}_{P}{}^{-1}$, which yielded a graph of relationships among individuals. Indeed, a zero *ij*-element in ${\tilde{G}}_{P}{}^{-1}$ indicates that individuals *i* and *j* are unrelated conditionally on all other individuals, which corresponds to no edge between individuals *i* and *j* in the underlying graph of recent relationships.

For a given sample covariance matrix Σ, the graphical LASSO infers a sparse precision matrix $\Sigma^{-1}$ by maximizing the Gaussian likelihood of the data (as represented by Σ), penalized by a *L_1_*-norm penalty $\lambda {\left\|\Sigma^{-1}\right\|}_{1}$, where *λ* is the regularization parameter and ${\left\|\Sigma^{-1}\right\|}_{1}$ is the sum of absolute values in $\Sigma^{-1}$ (Friedman *et al.* 2008).

In this study, regularization of $G_{P}$ was performed as follows:

1. Standardizing $G_{P}$ to obtain the corresponding correlation matrix $\Gamma_{P}$: $\Gamma_{P}=\text{diag}{\left(G_{P}\right)}^{-1/2}G_{P}\text{diag}{\left(G_{P}\right)}^{-1/2}$
2. Applying the graphical LASSO algorithm to $\Gamma_{P}$, to obtain the regularized correlation matrix ${\tilde{\Gamma }}_{P}$
3. Rescaling $\Gamma_{P}$ to obtain ${\tilde{G}}_{P}=\text{diag}{\left(G_{P}\right)}^{1/2}{\tilde{\Gamma }}_{P}\text{diag}{\left(G_{P}\right)}^{1/2}$

The graphical LASSO algorithm was run using the R package huge (Zhao *et al.* 2012). The regularization parameter *λ* was chosen to maximize the restricted likelihood for the following model:

$$y=1_{n}\mu +u+e;\;\left[\begin{array}{c} u\\ e \end{array}\right]∼N\left(\left[\begin{array}{c} 0\\ 0 \end{array}\right],\left[\begin{array}{cc} \tilde{G}\sigma_{u}^{2} & 0\\ 0 & I_{n}\sigma_{e}^{2} \end{array}\right]\right)$$

where **y** is the *n*-vector of genotype means at a given trait; $\tilde{G}=PP^{'}+{\tilde{G}}_{P}$, depending on *λ*, consists of regularized relationships; $\sigma_{u}^{2}$ is the variance of breeding values (here not equal to the variance of marker effects due to scaling and regularization on $\tilde{G}$); $1_{n}\mu$ is the *n*-vector of intercept values; $I_{n}\sigma_{e}^{2}$ is the covariance matrix for errors considered independent and identically distributed. Here we chose the value of *λ* for which the corresponding matrix $\tilde{G}$ resulted in the highest restricted likelihood for the aforementioned model fitted to the whole sample. Possible values of *λ* were the *q*-quantiles of absolute values in $\Gamma_{P}$, with *q* varying from 0.05 to 1 by step of 0.05. The restricted likelihood as a function of *λ* depended on **y**, so optimization of *λ* was performed for each trait separately.

## Appendix 2

### Multi-population GBLUP models for heterogeneous calibration sets

In this section, $1_{t}$, $I_{t}$, and $J_{t}$ refer to the vector of ones, identity matrix, and matrix of ones, respectively, of dimensions t, t × t and t × t (where *t* is specified).

Consider the following model for population-specific marker effects with respect to *K* populations and *n* genotypes:

$$\bar{y}=\bar{\text{Q}}\alpha +\bar{X}\bar{\beta }+\bar{e};\;\left[\begin{array}{c} e¯\\ \beta ¯\\ y¯ \end{array}\right]∼N\left(\left[\begin{array}{c} 0\\ 0\\ \bar{\text{Q}}\alpha \end{array}\right],\left[\begin{array}{ccc} R¯ & 0 & R¯\\ 0 & \left(\Omega_{K}⊗I_{m}\right)\sigma_{\beta }^{2} & \left(\Omega_{K}⊗I_{m}\right){X¯}^{\prime }\sigma_{\beta }^{2}\\ R¯ & X¯\left(\Omega_{K}⊗I_{m}\right)\sigma_{\beta }^{2} & \bar{V} \end{array}\right]\right);\;\bar{V}=\bar{X}\left(\Omega_{K}⊗I_{m}\right){\bar{X}}^{\prime }\sigma_{\beta }^{2}+\bar{R}$$

where ⊗ indicates the Kronecker product; $\bar{\text{Q}}=\left(1_{K}⊗\text{Q}\right)$ is the *Kn* × *p* design matrix for the *p*-vector α of fixed effects; $\bar{X}=\left(I_{K}⊗X\right)$ is the *Kn* × *Km* marker-data matrix for the *Km*-vector $\bar{\beta }$ of marker effects at each of the *K* populations, with variance $\left(\Omega_{K}⊗I_{m}\right)\sigma_{\beta }^{2}$. The matrix $\Omega_{K}$ reflects covariances in marker effects between populations. The *Kn*-vector $\bar{y}$, containing the phenotypic values for the *n* genotypes at each of the *K* populations, is hypothetical (and ill-defined from a practical standpoint), since genotypes typically do not belong to more than one population. The *Kn*-vector of residuals $\bar{e}$, with unspecified variance $\bar{R}=\left(I_{K}⊗R\right)$, is assumed to be uncorrelated to marker effects $\bar{\beta }$.

Let $\bar{u}=\bar{X}\bar{\beta }$ be the *Kn*-vector of additive genetic effects at each of the *K* populations, as a linear combination of a normally-distributed vector ($\bar{\beta }$), $\bar{u}$ follows a normal distribution with expectation and variance as follows (Lehermeier *et al.*, 2015):

$$\text{E}\left[\bar{u}\right]=\bar{X}\text{E}\left[\bar{\beta }\right]=0$$

$$\text{Var}\left[\bar{u}\right]=\bar{X}\left(\Omega_{K}⊗I_{m}\right){\bar{X}}^{\prime }\sigma_{\beta }^{2}=\left(I_{K}⊗X\right)\left(\Omega_{K}⊗I_{m}\right)\left(I_{K}⊗X^{\prime }\right)\sigma_{\beta }^{2}=\left(\Omega_{K}⊗X\right)\left(I_{K}⊗X^{\prime }\right)\sigma_{\beta }^{2}=\left(\Omega_{K}⊗XX^{\prime }\right)\sigma_{\beta }^{2}$$

So a multi-population model for breeding values that is equivalent to the model described above, by identical mean and variance structures, is as follows:

$$\bar{y}=\bar{\text{Q}}\alpha +\bar{u}+\bar{e};\;\left[\begin{array}{c} e¯\\ u¯\\ y¯ \end{array}\right]∼N\left(\left[\begin{array}{c} 0\\ 0\\ \bar{\text{Q}}\alpha \end{array}\right],\left[\begin{array}{ccc} R¯ & 0 & R¯\\ 0 & \left(\Omega_{K}⊗XX^{\prime }\right)\sigma_{\beta }^{2} & \left(\Omega_{K}⊗XX^{\prime }\right)\sigma_{\beta }^{2}\\ R¯ & \left(\Omega_{K}⊗XX^{\prime }\right)\sigma_{\beta }^{2} & \bar{V} \end{array}\right]\right)$$

Now assume that K=n, and each population corresponds to the specific genetic background of each individual separately. By considering only observations at every individual’s specific genetic background, the above model reduces to:

$$y=\text{Q}\alpha +u+e;\left[\begin{array}{c} e\\ u\\ y \end{array}\right]∼N\left(\left[\begin{array}{c} 0\\ 0\\ \text{Q}\alpha \end{array}\right],\left[\begin{array}{ccc} R & 0 & R\\ 0 & \left(\Omega_{n}○XX^{\prime }\right)\sigma_{\beta }^{2} & \left(\Omega_{n}○XX^{\prime }\right)\sigma_{\beta }^{2}\\ R & \left(\Omega_{n}○XX^{\prime }\right)\sigma_{\beta }^{2} & V \end{array}\right]\right);$$

$$V=\left(\Omega_{n}○XX^{\prime }\right)\sigma_{\beta }^{2}+R$$

where ○ is the Hadamard product; y is the typical *n*-vector of observed phenotypic values; u and e are the corresponding additive genetic effects and residuals, respectively. Individual-specific marker effects are therefore accounted for by multiplying each element of the relationship matrix $XX^{\prime }$ by the corresponding element of $\Omega_{n}$, thereby reflecting correlations in marker effects among individuals’ genetic backgrounds.

In general, we propose to infer $\Omega_{n}={\left(\omega_{ij}\right)}_{n\times n}$ by $\omega_{ij}=\kappa \left(\phi \left(x_{i}\right),\phi \left(x_{j}\right)\right)$, where ϕ is some function of the *m*-vectors of marker variables $x_{i}$ and $x_{j}$, for any pair of individuals *i* and *j*, and *κ* is a valid kernel function guaranteeing that $\Omega_{n}$ be positive semi-definite. In *MPM-Mixture*, $\phi \left(x_{i}\right)=a_{i}$ (*K*-vector of admixture coefficients for *i*) for any individual *i*, and the kernel function is $\kappa_{\rho }\left(a_{i},a_{j}\right)=\rho a_{i}^{\prime }a_{j}+(1-\rho )$, so that $\Omega_{n}$ is a “mixture” between a matrix of correlations restricted to population clusters and a matrix allowing full exchange of information across clusters, as in a standard GBLUP model. More generally, one could define the kernel function as $\kappa_{\rho ,\Theta }\left(a_{i},a_{j}\right)=\rho a_{i}^{\prime }\Theta_{K}a_{j}+(1-\rho )$, where $\Theta_{K}$ is a *K* × *K* matrix depicting relationships among clusters. Here, we simply set $\Theta_{K}=I_{K}$ and adjusted the kernel function (by restricted maximum likelihood) for *ρ* only.

In *MPM-Matérn*, $\phi \left(x_{i}\right)=p_{i}$ (*d*-vector of PC coordinates for *i*) for any individual *i*, and the kernel function is a Matérn function $\kappa_{\nu ,h}\left(p_{i},p_{j}\right)$ of ${\left\|p_{i}-p_{j}\right\|}_{2}$, where ${\left\|.\right\|}_{2}$ is the Euclidean norm. Notably, it can be shown that ${\left\|p_{i}-p_{j}\right\|}_{2}$ is proportional to ${\left\|\pi_{P}{}_{i}-\pi_{P}{}_{j}\right\|}_{2}$, where $\pi_{P}{}_{i}$ ($\pi_{Pj}$) is the *m*-vector of individual-specific allele frequencies for individuals *i* (*j*), defined by projection of matrix **X** onto the column space of ${\text{Q}}_{P}=\left[\begin{array}{cc} 1_{n} & P \end{array}\right]$ (Appendix 3). So ${\left\|p_{i}-p_{j}\right\|}_{2}$, which reflects differentiation with respect to coordinates at the leading PCs of **X**, also reflects differentiation with respect to individual-specific allele frequencies, with an underlying population structure represented by the same PCs. The allele frequencies $\pi_{P}{}_{i}$ have been introduced by Conomos *et al.* (2016), in a study where they also recommended using principal components from a subset of unrelated individuals in **X**. Here, we simply applied PCA on the whole matrix **X**.

## Appendix 3

### Relationship between Euclidean distance based on principal components and Euclidean distance based on individual-specific allele frequencies

In this section, $1_{t}$, $I_{t}$, $J_{t}$ and $0_{s\times t}$ refer to the vector of ones, identity matrix, matrix of ones and matrix of zeros, respectively, of dimensions t, t × t, t × t and s × t (where *s* and *t* are specified).

We will consider the case where the PC matrix **P** consists of the first *d* PCs of **X**, and individual-specific allele frequencies are defined as (Conomos *et al.* 2016):

$$\Pi_{P}=\frac{1}{2}{\text{Q}}_{P}{\left({\text{Q}}_{P}{}^{\prime }{\text{Q}}_{P}\right)}^{-1}{\text{Q}}_{P}{}^{\prime }X$$

where ${\text{Q}}_{P}=\left[\begin{array}{cc} 1_{n} & P \end{array}\right]$ represents population structure through an intercept and the effects of the first *d* PCs of **X**. Vector $\pi_{P}{}_{i}$ ($\pi_{P}{}_{j}$) then consists of individual-specific allele frequencies (with respect to ${\text{Q}}_{P}$) for individual *i* (*j*), such that:

$$\pi_{P}{}_{i}=\frac{1}{2}q_{i}^{\prime }{\left({\text{Q}}_{P}{}^{\prime }{\text{Q}}_{P}\right)}^{-1}{\text{Q}}_{P}{}^{\prime }X$$

and similarly for $\pi_{Pj}$ ($q_{i}$ refers to the (d+1)-vector of population-structure variables from ${\text{Q}}_{P}$ for individual *i*).

We will show that ${\left\|p_{i}-p_{j}\right\|}_{2}=2{\left\|\pi_{P}{}_{i}-\pi_{P}{}_{j}\right\|}_{2}$ for any pair (*i*, *j*), *i.e.*, Euclidean distances based on *d* PCs are equivalent, by proportionality, to those based on *m* individual-specific allele frequencies, with such frequencies as defined above.

$${\left\|p_{i}-p_{j}\right\|}_{2}=\sqrt{\left(p_{i}^{\prime }-p_{j}^{\prime }\right)\left(p_{i}-p_{j}\right)}=\sqrt{p_{i}^{\prime }p_{i}+p_{j}^{\prime }p_{j}-2p_{i}^{\prime }p_{j}}$$

$${\left\|\pi_{P}{}_{i}-\pi_{P}{}_{j}\right\|}_{2}=\sqrt{\pi_{P}{}_{i}^{\prime }\pi_{P}{}_{i}+\pi_{P}{}_{j}^{\prime }\pi_{P}{}_{j}-2\pi_{P}{}_{i}^{\prime }\pi_{P}{}_{j}}=\frac{1}{2}\sqrt{q_{i}^{\prime }Mq_{i}+q_{j}^{\prime }Mq_{j}-2q_{i}^{\prime }Mq_{j}}$$

where $M={\left({\text{Q}}_{P}{}^{\prime }{\text{Q}}_{P}\right)}^{-1}{\text{Q}}_{P}{}^{\prime }XX^{'}{\text{Q}}_{P}{\left({\text{Q}}_{P}{}^{\prime }{\text{Q}}_{P}\right)}^{-1}$.

Below, we will specify **M** more explicitly, to subsequently show that ${\left\|p_{i}-p_{j}\right\|}_{2}=2{\left\|\pi_{P}{}_{i}-\pi_{P}{}_{j}\right\|}_{2}$.

Let $\left(I_{n}-\frac{J_{n}}{n}\right)X$ be the matrix of marker variables centered around their respective overall mean. Assuming m≥n, by eigenvalue decomposition $\left(I_{n}-\frac{J_{n}}{n}\right)XX^{'}\left(I_{n}-\frac{J_{n}}{n}\right)=U\Lambda U^{'}$, with **U** the n ×n matrix of eigenvectors and Λ the n×n diagonal matrix of eigenvalues of $\left(I_{n}-\frac{J_{n}}{n}\right)XX^{'}\left(I_{n}-\frac{J_{n}}{n}\right)$; and $P=U_{d}\Lambda_{d}^{1/2}$, where $U_{d}$ is the n×d matrix of leading eigenvectors and $\Lambda_{d}^{1/2}$ is the d×d diagonal matrix of corresponding singular values, assumed strictly positive.

Because $U_{d}$ consist of left-eigenvectors of a column-centered matrix (associated with strictly positive eigenvalues), $U_{d}^{\prime }1_{n}=0_{d\times 1}$ so $P^{\prime }1_{n}=\Lambda_{d}^{1/2}U_{d}^{\prime }1_{n}=0_{d\times 1}$.

Besides, $P^{\prime }P=\Lambda_{d}$.

So:

$${\left({\text{Q}}_{P}{}^{\prime }{\text{Q}}_{P}\right)}^{-1}={\left[\begin{array}{cc} 1_{n}^{\prime }1_{n} & 1_{n}^{\prime }P\\ P^{\prime }1_{n} & P^{\prime }P \end{array}\right]}^{-1}=\left[\begin{array}{cc} n^{-1} & 0_{1\times d}\\ 0_{d\times 1} & \Lambda_{d}^{-1} \end{array}\right]$$

Moreover ${\text{Q}}_{P}^{\prime }X=\left[\begin{array}{c} 1_{n}^{\prime }\\ P^{\prime } \end{array}\right]\left(\left(I_{n}-\frac{J_{n}}{n}\right)X+\frac{J_{n}}{n}X\right)=\left[\begin{array}{c} 1_{n}^{\prime }X\\ P^{\prime }\left(I_{n}-\frac{J_{n}}{n}\right)X \end{array}\right]$ because $P^{\prime }J_{n}=\left(P^{\prime }1_{n}\right)1_{n}^{\prime }=0_{d\times n}$.

So

$${\left({\text{Q}}_{P}{}^{\prime }{\text{Q}}_{P}\right)}^{-1}{\text{Q}}_{P}^{\prime }X=\left[\begin{array}{c} \mu^{'}\\ \Lambda_{d}^{-1/2}U_{d}^{\prime }\left(I_{n}-\frac{J_{n}}{n}\right)X \end{array}\right]$$

where $\mu^{'}=\frac{1}{n}1_{n}^{\prime }X$

Finally, $M=\left({\left({\text{Q}}_{P}{}^{\prime }{\text{Q}}_{P}\right)}^{-1}{\text{Q}}_{P}{}^{\prime }X\right)\left(X^{'}{\text{Q}}_{P}{\left({\text{Q}}_{P}{}^{\prime }{\text{Q}}_{P}\right)}^{-1}\right)=\left[\begin{array}{cc} \mu^{'}\mu & a^{\prime }\\ a & I_{d} \end{array}\right]$

with:

$$\Lambda_{d}^{-1/2}U_{d}^{\prime }\left(I_{n}-\frac{J_{n}}{n}\right)XX^{'}\left(I_{n}-\frac{J_{n}}{n}\right)U_{d}\Lambda_{d}^{-1/2}=\Lambda_{d}^{-1/2}\left(U_{d}^{\prime }U\Lambda U^{'}U_{d}\right)\Lambda_{d}^{-1/2}=\Lambda_{d}^{-1/2}\Lambda_{d}\Lambda_{d}^{-1/2}=I_{d}$$

$$a=\Lambda_{d}^{-1/2}U_{d}^{\prime }\left(I_{n}-\frac{J_{n}}{n}\right)X\mu$$

Therefore, for any pair of individuals (*i*, *j*):

$$q_{i}^{\prime }Mq_{j}=\left[\begin{array}{cc} 1 & p_{i}^{\prime } \end{array}\right]M\left[\begin{array}{c} 1\\ p_{j} \end{array}\right]=\mu^{'}\mu +p_{i}^{\prime }a+a^{\prime }p_{j}+p_{i}^{\prime }p_{j}$$

So:

$$2{\left\|\pi_{P}{}_{i}-\pi_{P}{}_{j}\right\|}_{2}=\sqrt{q_{i}^{\prime }Mq_{i}+q_{j}^{\prime }Mq_{j}-2q_{i}^{\prime }Mq_{j}}=\sqrt{\left(\mu^{'}\mu +2p_{i}^{\prime }a+p_{i}^{\prime }p_{i}\right)+\left(\mu^{'}\mu +2p_{j}^{\prime }a+p_{j}^{\prime }p_{j}\right)-2\mu^{'}\mu -2p_{i}^{\prime }a-2a^{\prime }p_{j}-2p_{i}^{\prime }p_{j}}=\sqrt{p_{i}^{\prime }p_{i}+p_{j}^{\prime }p_{j}-2p_{i}^{\prime }p_{j}}={\left\|p_{i}-p_{j}\right\|}_{2}$$
